# Understanding the basics and clinical applications of contrast-enhanced ultrasound for breast lesions

**DOI:** 10.1007/s10396-024-01502-3

**Published:** 2024-10-24

**Authors:** Toshikazu Ito, Yoshifumi Komoike

**Affiliations:** Division of Breast and Endocrine Surgery Department of Surgery, Kindai University Faculty of Medicine, Osaka, Japan

In 2020, breast cancer surpassed lung cancer as the most common cancer worldwide, with an estimated 2.3 million new cases, accounting for 11.7% of all cancer diagnoses [1]. Breast density is a recognized risk factor for breast cancer, with higher breast density being associated with an increased risk of developing the disease. Asian women are generally observed to have higher breast density compared to women of other ethnicities, which can reduce the accuracy of mammography screening as a standalone diagnostic tool. In Asia, the age-specific incidence of female breast cancer peaks between the ages of 40 and 49 years, whereas in Western countries, the peak incidence occurs between 60 and 70 years [2]. Breast cancer remains one of the leading causes of cancer-related mortality among women worldwide. Therefore, early diagnosis and prompt treatment of breast cancer are critical for reducing mortality rates and improving overall prognosis.

We are pleased to present a comprehensive review on the use of contrast-enhanced ultrasound (CEUS) for evaluation of breast lesions. This special issue aims to provide readers with a thorough understanding of the principles underlying CEUS, its diagnostic role in breast imaging, and its various clinical applications. Additionally, the review covers foundational information on angiogenesis inhibitors, offering insights into tumor angiogenesis, which is closely linked to the diagnostic utility of CEUS.

In recent years, the accessibility of testing for hereditary breast and ovarian cancer syndrome (HBOC) has improved, leading to a clearer understanding of its clinical features. Contrast-enhanced magnetic resonance imaging (MRI) is recommended for the diagnosis of breast cancer in patients with BRCA mutations [3]. However, certain patients, including those with allergies to contrast agents, impaired renal function, or the presence of metal implants, may face challenges in undergoing contrast-enhanced MRI.

A meta-analysis comparing conventional B-mode ultrasound (US) and CEUS revealed that the pooled sensitivity and specificity of B-mode US were 89.6% and 60.9%, respectively. However, with the addition of CEUS, these values improved to 95.1% and 80.9%. Based on these findings, the 2022 edition of the Japanese Breast Cancer Society Clinical Practice Guidelines for Breast Cancer Screening and Diagnosis recommended the use of CEUS for differentiating between benign and malignant breast lesions [4].

CEUS has diagnostic capabilities comparable to those of contrast-enhanced breast MRI. It offers a simpler imaging technique that allows the patient to remain in a more comfortable position and can be completed in a relatively short time. Furthermore, CEUS has the advantage of being safe for patients with contrast agent allergies, renal dysfunction, or metal implants [5].

While the diagnosis of typical breast cancer presenting as a mass is relatively straightforward, accurately assessing the extent of breast cancer, such as intraductal spread, remains a challenge. Diagnosing non-mass abnormalities or lesions, such as hypoechoic areas in the mammary gland, ductal abnormalities, architectural distortions, or multiple small cysts, will become increasingly important [6].

The use of CEUS in diagnosis is expected to enhance the accuracy of detecting these types of lesions. There are three primary risk factors for breast cancer: genetic predisposition, individual predisposition, and pathological histological factors. Among the pathological histological factors, conditions such as atypical ductal hyperplasia (ADH), atypical lobular hyperplasia (ALH), and lobular carcinoma in situ (LCIS) are considered to confer a high risk of progression to breast cancer. Reports suggest that the upgrades of LCIS and ductal carcinoma in situ (DCIS) can be assessed using CEUS [7].

Compared to Western populations, Japanese women tend to have denser breasts. As demonstrated by the Japan Strategic Anti-cancer Randomized Trial (J-START), the role of US is significant, supporting the rationale for combining mammography (MG) with US for comprehensive breast cancer assessment [8]. The J-START reported that the combination of ultrasound screening in relatively younger women in their 40 s facilitates the early detection of breast cancer. Given that the incidence rate peaks between the ages of 40 and 49, ultrasound screening may also offer substantial benefits for BRCA mutation carriers.

Mine et al. discussed the fundamental characteristics and administration methods of Sonazoid, a contrast agent used in breast imaging, while also elucidating the principles behind imaging techniques in CEUS. They provided an overview of the imaging principles used in CEUS examinations, including filter techniques, pulse inversion, amplitude modulation, and amplitude modulation pulse inversion. Specifically, they explained imaging techniques that utilize pulse inversion and amplitude modulation, which are commonly employed in breast imaging, presenting both simulation results and experimental data [9].

In 2002, Omoto et al. developed a novel method for sentinel lymph node identification using albumin as an ultrasound contrast agent (UCA) in animal experiments. By 2006, they successfully demonstrated the ability to identify lymph nodes in breast cancer patients. Subsequently, they conducted a multicenter study on sentinel lymph node identification using Sonazoid as a tracer in breast cancer patients, reporting favorable results. In this special issue, Omoto et al. presented not only the basic and clinical research findings from their institution on breast cancer but also detailed the specific methods and results of sentinel lymph node identification using various UCAs across different fields [10].

Accurate diagnosis of lymph node metastasis in breast cancer patients is critical for prognostic assessment and treatment planning. Preoperative imaging modalities typically include conventional US, MRI, computed tomography (CT), and positron emission tomography/computed tomography (PET/CT). While conventional US is widely utilized due to its high resolution and sensitivity, it poses challenges such as operator dependency and difficulty in detecting small metastatic lymph nodes [11].

Mori et al. investigated the role of US in diagnosing liquefied lymph node metastasis and compared its performance with other diagnostic modalities. They also explored the anatomical structure of the axillary region and lymph nodes, providing a clear explanation of the two categories of CEUS techniques used in axillary lymph node evaluation. These categories include the identification of sentinel lymph nodes via percutaneous contrast agent injection through the lymphatic vessels and the diagnosis of lymph node metastasis via subcutaneous contrast agent injection (lymphatic CEUS), as well as diagnosis through intravenous contrast agent injection (perfusion CEUS) [12].

In preoperative contrast-enhanced MRI examinations for breast cancer, approximately 20–30% of cases are found to have incidental breast lesions that are not detected on MG or conventional US. Miyake et al. explained, based on their research findings, that even when MRI-detected lesions are difficult to identify using second-look US, the use of third-look CEUS facilitates effective detection and diagnosis of these lesions. The combined use of CEUS and needle biopsy is anticipated to reduce the frequency of MRI-guided biopsies [13].

In this special feature, Hida et al. provided a comprehensive review of anti-angiogenic agents used across various fields, including bevacizumab, a monoclonal antibody targeting human vascular endothelial growth factor (VEGF), along with detailed insights into tumor vasculature. They also discussed the mTOR pathway, which plays a crucial role in angiogenesis, cell proliferation, and metabolism, as well as the therapeutic potential of mTOR inhibitors. Furthermore, they highlighted how anti-angiogenic agents enhance the efficacy of both chemotherapy and immunotherapy and discussed the unique characteristics of tumor vascular endothelial cells [14].

In 1971, Folkman first introduced the concept of cancer treatment through the inhibition of angiogenesis after identifying angiogenic factors [15]. The balance between multiple angiogenic factors and endogenous angiogenesis inhibitors plays a critical role in the regulation of angiogenesis, with an excess of angiogenic factors in cancer leading to enhanced angiogenic activity.

VEGF is recognized as a key inflammatory cytokine and regulator of angiogenesis, serving as a pivotal driver of blood vessel growth and vascular permeability [16, 17]. VEGF-A, a well-known angiogenic factor, has its gene expression stimulated by hypoxia, growth signals, and tumor suppressor genes.

Most existing angiogenesis inhibitors target VEGF, its receptor (VEGFR), and associated signaling pathways. Tumor blood vessels are characterized by irregular distribution, tortuous morphology, uneven vessel diameters, and inconsistent vessel density, leading to a disrupted hierarchical structure of arteries, capillaries, and veins. In cancers with active angiogenesis, the immature and highly permeable blood vessels often result in poor circulation, contributing to hypoxia within the tumor tissue [18). The response to anti-angiogenic therapy can be assessed through architectural imaging of tumor vasculature, and CEUS serves as a valuable tool in enhancing diagnostic accuracy [19].

While the sensitivity of conventional US in differentiating between benign and malignant breast masses is already high, the addition of CEUS has shown further improvements in sensitivity. Moreover, the use of CEUS has enhanced specificity, reducing the number of unnecessary biopsies for breast lesions [5].

Ito et al. discussed the history of CEUS and highlighted the differences between CEUS applications in the breast and liver regions. They provided a detailed explanation of the diagnostic criteria for CEUS in breast lesions, dividing their analysis into qualitative and quantitative evaluation methods. Additionally, they addressed the current status of CEUS diagnosis for non-mass abnormalities, noting that specific diagnostic criteria have not yet been established. The paper also discussed pathological prognostic factors, assessment of treatment efficacy in preoperative chemotherapy, and evaluation methods for CEUS in lymph node analysis [20].

While there is still a need to accumulate further data on breast CEUS, it is anticipated that CEUS will improve diagnostic accuracy, particularly for small lesions that are challenging to diagnose with MG or conventional US, as well as in cases related to HBOC or non-mass lesions.

## Conclusion

We have invited experts to provide in-depth explanations of the principles, methods, clinical applications, and the current status of tumor angiogenesis and anti-angiogenic agents in the context of CEUS diagnosis for breast lesions. By establishing a common understanding of this project's content, it will serve as a valuable reference for writing presentations and research papers on breast CEUS and angiogenesis.

## Additional

Different terminologies may be used to describe similar findings in various papers. In this special issue, we have respected the originality of each author by preserving their original terminology. This does not detract from the academic value of the individual papers or the project as a whole.

## References

[1] Sung H, Ferlay J, MSc, Rebecca L. Siegel RL, Global cancer statistics, et al. GLOBOCAN estimates of incidence and mortality worldwide for 36 cancers in 185 countries. CA Cancer J Clin. 2020;2021:209–49. 10.3322/caac.21660 33538338

[2] Ishihara S, Taira N, Kawasaki K, et al. Association between mammographic breast density and lifestyle in Japanese women. Acta Med Okayama. 2013;67:145–51. 23804137 10.18926/AMO/50407

[3] Tozaki M, Nakamura S. Current status of breast cancer screening in high-risk women in Japan. Breast Cancer. 2021;28:1181–7. 32627143 10.1007/s12282-020-01103-1

[4] Kubota K, Nakashima K, Nakashima K, et al. The Japanese breast cancer society clinical practice guidelines for breast cancer screening and diagnosis. Breast Cancer. 2024. 10.1007/s12282-023-01521-x. 39138789 10.1007/s12282-024-01623-0 PMC11489246

[5] Miyamato Y, Ito T, Takada E, et al. Efficacy of sonazoid (perflubutane) for contrast-enhanced ultrasound in the differentiation of focal breast lesions: phase 3 multicenter clinical trial. AJR. 2014;202:W400–7. 24660739 10.2214/AJR.12.10518

[6] Ito T, Ueno E, Endo T, et al. The Japan society of ultrasonics in medicine guidelines on non-mass abnormalities of the breast. J Med Ultrason. 2023;50:331–9. 10.1007/s10396-023-01308-9 PMC10354171 37261555

[7] Zhu Y, Jia X, Zhan W, et al. Adding contrast-enhanced ultrasound can improve the predictive ability of breast conventional ultrasound and mammography for pathological upgrade of biopsy-confirmed ductal carcinoma in situ. Eur J Radiol. 2024;180: 111687. 39213762 10.1016/j.ejrad.2024.111687

[8] Ohuchi N, Suzuki A, Sobue T, et al. Sensitivity and specifcity of mammography and adjunctive ultrasonography to screen for breast cancer in the Japan Strategic Anti-cancer Randomized Trial (J-START): a randomised controlled trial. Lancet. 2016;387:341–8. 26547101 10.1016/S0140-6736(15)00774-6

[9] Mine Y, Etsuo Takada E, Sugimoto K, et al. Principle of contrast-enhanced ultrasonography. J Med Ultra. 2024. 10.1007/s10396-024-01443-x. 10.1007/s10396-024-01443-x PMC11499413 38780871

[10] Omoto K, Kazushige Futsuhara K, Watanabe T. Sentinel lymph node identifcation using contrast-enhanced ultrasound in breast cancer: review of the literature. J Med Ultrason. 2024. 10.1007/s10396-024-01443-x. 10.1007/s10396-023-01313-y PMC11499350 37423960

[11] Mori N, Mugikura S, Miyashita M, et al. Perfusion contrastenhanced ultrasound to predict early lymph-node metastasis in breast cancer. Jpn J Radiol. 2019;37:145–53. 30460444 10.1007/s11604-018-0792-6

[12] Mori N, Li L, Matsuda M. Prospects of perfusion contrast-enhanced ultrasound (CE-US) in diagnosing axillary lymph node metastases in breast cancer: a comparison with lymphatic CE-US. J Med Ultrason. 2024. 10.1007/s10396-024-01444-w. 10.1007/s10396-024-01444-w PMC11499517 38642268

[13] Miyake T, Shimazu K. Third-look contrast-enhanced ultrasonography plus needle biopsy for diferential diagnosis of magnetic resonance imaging-only detected breast lesions. J Med Ultrason. 2023. 10.1007/s10396-023-01298-8. 10.1007/s10396-023-01298-8 PMC11499426 36905491

[14] Hida K, Maishi N, Matsuda A, et al. Beyond starving cancer: anti-angiogenic therapy. J Med Ultrason. 2023. 10.1007/s10396-023-01310-1. 10.1007/s10396-023-01310-1 PMC11499530 37170042

[15] Folkman J. Tumor angiogenesis: therapeutic implications. N Engl J Med. 1971;285:1182–6. 4938153 10.1056/NEJM197111182852108

[16] Motzer RJ, Penkov K, Haanen J, et al. Avelumab plus axitinib versus sunitinib for advanced renal-cell carcinoma. N Engl J Med. 2019;380:1103–15. 30779531 10.1056/NEJMoa1816047 PMC6716603

[17] Weis SM, Cheresh DA. Pathophysiological consequences of VEGF-induced vascular permeability. Nature. 2005;437:497–504. 16177780 10.1038/nature03987

[18] Folkman J. Anti-angiogenesis: new concept for therapy of solid tumors. Ann Surg. 1972;175:409–16. 5077799 10.1097/00000658-197203000-00014 PMC1355186

[19] Chen M, Wang WP, Jin WR, et al. Three-dimensional contrast-enhanced sonography in the assessment of breast tumor angiogenesis: correlation with microvessel density and vascular endothelial growth factor expression. J Ultrasound Med. 2014;33:835–46. 24764339 10.7863/ultra.33.5.835

[20] Ito T, Manabe H, Kubota M, et al. Current status and future perspectives of contrast-enhanced ultrasound diagnosis of breast lesions. J Med Ultrason. 2024. 10.1007/s10396-024-01486-0. 10.1007/s10396-024-01486-0 PMC11499542 39174799

